# An Observational Study of Regulatory Violations Related to Online Tobacco Product Marketing and Retailer Responses to US FDA Warning Letters

**DOI:** 10.1177/1179173X241300825

**Published:** 2024-11-13

**Authors:** Dorie E. Apollonio, Cathi E. Dennehy, Candy Tsourounis, Tanner Wakefield

**Affiliations:** 1 School of Pharmacy, 8785 University of California San Francisco, San Francisco, CA USA; 2 School of Medicine, 8785 University of California San Francisco, San Francisco, CA USA

**Keywords:** adolescent, electronic nicotine delivery systems, tobacco, marketing, COVID-19

## Abstract

**Background:**

Marketing of flavored tobacco products has drawn concern because they are used disproportionately by young people. Online retailers have marketed e-cigarette liquids and devices to minors despite knowing it is illegal. The onset of the COVID-19 pandemic resulted in substantial increases in online purchasing, however, there has been limited study of possible shifts in online tobacco product marketing associated with this change.

**Objectives:**

We sought to identify types of tobacco regulatory violations in 2021-2022, marketing claims made by retailers, the extent to which retailers had processes in place to deter minors from browsing websites, and the types of flavors promoted.

**Design and Methods:**

Our observational study was based on an initial sample of 100 tobacco retailers that had received FDA Warning Letters in 2020-2021. Using methods validated in previous research, we coded the letters for retailer information, violation type, and for retailers with an online presence, the types of products sold, and their marketing claims.

**Results:**

Most retailers with violations were in the US South (48%), 65% had an online presence at the time of analysis, and 53% had a website that offered online product sales. The most common type of violation was the sale of new tobacco products without required marketing authorization (83%). For the retailers in the sample with active websites, 42% were still selling a product that the FDA had indicated was marketed unlawfully. Among these retailers with active websites, 32% did not have processes in place to deter access by minors. Advertised flavors focused on food (eg, mango, honey) and concepts (eg, “love”, “classic”).

**Conclusions:**

Online retailers appeared less likely to remediate tobacco product violations identified by the FDA after the onset of the COVID-19 pandemic than before it, and few websites had strong processes in place that would deter youth browsing.

## Key Messages

### What is Already Known on This Topic

• Young people who use tobacco products disproportionately use flavored products, many of which are purchased online.
• Past research has found that retailers market e-cigarette liquids and devices to minors even after being notified that this is illegal.

### What This Study Adds

• This research considered shifts in online marketing by US retailers since the beginning of the COVID-19 pandemic, when online purchasing increased substantially.
How this study might affect research, practice, or policy
• Online tobacco retailers appeared less likely to remediate violations identified by the FDA after the onset of the COVID-19 pandemic than before it, and they did not have strong processes in place that would deter youth browsing.
• The FDA should consider expanding enforcement of existing regulations on tobacco products marketed unlawfully to reduce initiation of tobacco product use.

## Introduction

Historical studies of e-cigarette marketing in the US found that retailers focused on claims that alternative tobacco products could be used for cessation, were less expensive, and could be used in places where smoking was banned.^1-10^ Studies of e-cigarette marketing conducted in countries outside the US suggested a shift in messaging after 2016, finding that online e-cigarette retailers were increasingly placing social media links on their own sites, using cartoon images on products, promoting flavors, and doing little or nothing to verify age or to ensure products were not sold to minors.^11-16^

In 2016, FDA regulations undercut the ability to make certain advertising claims, specifically by deeming that all alternative tobacco products must operate under the same regulations as combustible cigarettes, including e-cigarettes and little cigars.^17,18^ These deeming regulations enabled enforcement against flavored products in 2020, indicating these products could not be marketed unless the sellers sought FDA approval.^19^ New tobacco products, including flavored e-cigarettes, were required to submit a Premarket Tobacco Product Application (PMTA) “that demonstrates a product is appropriate for the protection of public health.”^20^ This change may not have been sufficient to control inappropriate advertising; in 2021, the FDA requested information from four e-cigarette producers (Aspire, Joytech, Vaporesso, Voopoo) regarding the advertising of their products, in order to develop additional oversight.^21^

Flavored tobacco products have drawn regulatory and popular concern because they are attractive to young people. As of 2024, 7.8% of high school students in the US reported current use of e-cigarettes; of these, 87.6% used flavored products, the most popular of which were fruit, candy, desserts and other sweets, and mint.^22^ Flavored tobacco use positively correlates to dual and poly-tobacco use.^23,24^ In November 2018, the US Food and Drug Administration (FDA) issued a press announcement stating that e-cigarette marketing was so similar to candy and juices that children could mistake them for real food products.^25,26^ A 2019 systematic review of social media advertising noted increases in the promotion of tobacco product flavors.^27,28^

In 2017, 6.3% of tobacco products were purchased online^29^; in 2020, with the advent of the COVID-19 pandemic, online purchasing of all products increased dramatically.^30,31^ For tobacco products specifically, a small sample of US young adults in 2020 reported changes in tobacco product use related to the COVID-19 pandemic,^32^ and research in South Korea identified an increase in online tobacco sales in 2020, with specific promotions related to COVID-19 such as “Stay home and vape” as well as a shift toward marketing e-cigarettes as “trendy.”^33^ By 2020 one in five US youth and young adults (20%) had purchased e-cigarettes online, with 27.5% of those purchases involving no age verification.^34^ In 2021, one-third of underage users purchased e-cigarettes and other tobacco products online.^35^ Some online retailers market e-cigarette liquids and devices to minors despite knowing it is illegal; a study assessing online tobacco retailer responses to FDA Warning Letters issued in 2018 found that over 98% of violations were marketing e-cigarettes to minors, sales of e-cigarettes to minors, or both, and that 16.8% of retailers did not correct their violations after receiving warnings.^36^

Understanding retailer advertising has been recognized as critical to effective tobacco control.^4^ In this study we sought to assess potential shifts in marketing claims for tobacco products, focusing on e-cigarettes, by online retailers during the COVID-19 pandemic, when online purchasing of all products increased. Given that the FDA regulation had increasingly focused on flavors that appeal to children since 2016, we anticipated that retailers were no longer primarily marketing e-cigarettes for cessation or for use in places where smoking is banned. Our aims were to identify the types of regulatory violations during this period, the marketing claims made by retailers, the extent to which retailers had processes in place to deter access by minors, and the types of flavors promoted. Consistent with research conducted outside the US,^11-16^ we anticipated that retailers who had received regulatory warnings, even if they remediated specific violations identified by the FDA, were unlikely to request or validate age verification and used marketing known to appeal to underage purchasers, including promoting flavored products using food imagery. We focused on online retailers that had received FDA Warning Letters for regulatory violations because this source makes it possible to assess novel and illegal marketing strategies.^36^

## Methods

We conducted an observational study of online tobacco retailers that had received FDA Warning Letters in 2020-2021.

### Data Source and Collection

Consistent with methods validated in previous research, we used the online FDA Warning Letters database^37^ to identify tobacco-related marketing violations for 2020-2021.^36^ *Initial inclusion and exclusion criteria:* We filtered the database for letters categorized as “Center for Tobacco Products” to exclude pharmaceutical and other unrelated warnings. The FDA permits retailers up to 15 days to remediate violations, so database searches commenced in February 2022 to ensure that no retailer that had received a letter in 2021 was still in the window of time permitted by the FDA for remediation. *Sampling:* The Center for Tobacco Products issued 139 Warning Letters in 2020 and 289 Warning Letters in 2021. Using the random number function in Stata v17, we drew an initial sample of 100 retailers that had received FDA Warning Letters (23% of 2020-2021 letters); a sample size consistent with prior research on this topic.^36^ *Additional inclusion and exclusion criteria:* For any retailer that had received multiple Warning Letters, we kept the earliest letter in the sample and excluded later letters to ensure that our assessment considered retailers that had maximum time to remediate (Supplement, Table S1). We excluded warning letters related solely to combustible cigarettes and similar tobacco products (eg, cigars); most letters referenced e-cigarettes and accessories only, however we included retailers that sold other tobacco products or cannabis products in addition to e-cigarette products. *Timeline:* Coding was conducted between March and July 2022. All the retailers in the final sample had received letters at least 3 months prior to the start of the study.

### Measures

Our outcomes, as described above, included regulatory violations classified by type, marketing claims (binary variable for their presence or absence, and binary indicator variables for characteristics such as presence of the required health warning), the extent to which retailers had processes in place to deter access by minors (binary for each process, and a free text field for age if not 18 or 21 years), and the types of flavors, if any, promoted on websites (binary for presence or absence of a flavor description; specific descriptions were copied directly from product pages). We first characterized each letter by date of issue, name of the retailer, recipient named on the letter, websites when available, and contact information. Retailer geographic locations were classified using Census Regions and Divisions of the United States. We abstracted information from each letter regarding products named and the violations listed, including the number of violations (continuous variable), nature of violations (binary variable for each type), number and names of products identified (if any), whether the retailer had registered as a manufacturer (binary variable), and actions required by the FDA (copied directly from each letter text and organized by type).

Identifying the other outcomes (additional marketing claims, the extent to which retailers had processes in place to deter access by minors, and the types of flavors promoted on these websites) required accessing the retailer websites directly. Using the contact information or direct web address provided in the letter, we searched online for each retailer to determine whether a link to the retailer website, if provided, was still active and allowed visitors to enter without member log-in. For retailers whose web presence was not indicated in the FDA Warning Letter or was no longer active, we searched online for any separate online business webpage and, where available, coded these sites. For retailers with an online presence, we coded the characteristics of the landing page including the following variables: number of products and brands listed, types of products available binary variable for each product), seasonal specials (binary variable for presence or absence), links to social media (binary variable for presence or absence), the presence or absence of age-gating (binary variable) and if present, its characteristics (eg, age allowed for entry, click-through or other verification), whether the retailer continued to sell any or all of the products named in the FDA Warning Letter, and types, characteristics (flavors, taste, social acceptability, health warnings; together, these constituted marketing claims), and prices of those products. For each retailer, we noted the date we retrieved information and saved screenshots of their landing pages, age-gating (if any) and product pages. We used REDCap, a secure, web-based software platform designed to collect and manage study data^17,18^ to collect and code preliminary data.^38,39^

### Analytical strategy

Our coding and analysis built on an existing instrument developed in a previous study of FDA Warning Letters, which classified violations identified by the FDA by type (sales to a minor, relationship to food, use of cartoon imagery, imminent health consequences, other); because the data collection was completed after the deeming regulations, categorization also included PMTA violations.^36^ Additional characteristics (eg, flavors, health warnings) were coded as to whether these characteristics were specifically indicated in a marketing text for the relevant products. All investigators (DA, TW, CD, CT) pilot-tested the instrument by coding the first three Warning Letters as a group, then retested it by having pairs of investigators double-code 10 randomly selected retailer sites each. Upon review of these double-coded retailer sites, each pair had made the same coding decisions. As a result, from that point forward one author coded each retailer site individually. If an individual coded felt that any retailer claims appeared unclear, the site was reviewed in a weekly meeting of all the investigators until all four reached agreement on the appropriate classification.

We used Stata v17 for all quantitative analyses. We calculated descriptive statistics including the frequency and format (text, picture, and video) of different types of marketing claims. We assessed the most common violations and marketing claims made by online retailers in each category.^11-16^ We assessed the frequency and types of age verification on online retailer sites, anticipating that most retailers used click-through verification or no verification.^4,40^ We also identified the share of retailers that received FDA Warning Letters that had corrected violations by following the directive made by the FDA in the Warning Letter (for example, by no longer selling the product identified as being in violation).^36,41^ We classified flavors into categories, creating a complete list of all products identified in Warning Letters. All four authors reviewed the list together, while referencing contemporaneous research on flavors that identified “concept” categorizations.^42^

### Ethical Approval

The research was conducted using data that can be accessed freely by the public without special permission or application, and as a result was excluded from institutional review board assessment (UCSF IRB #10-01262).

## Results

**Figure 1.**
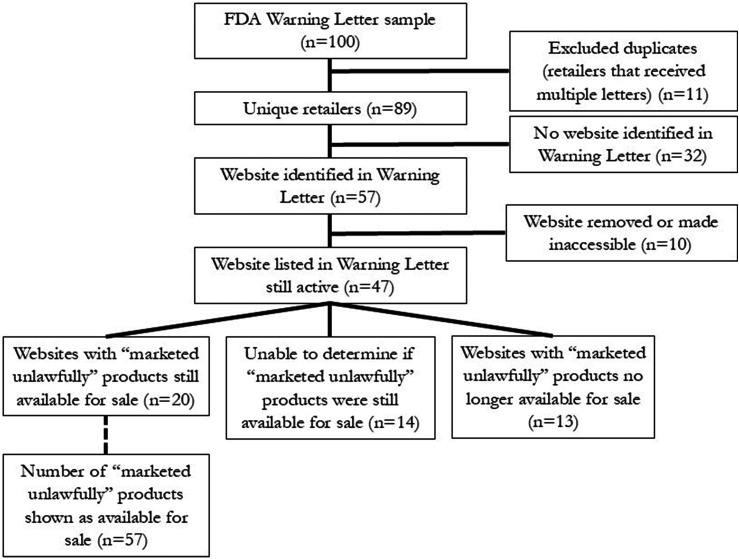
Flowchart of retailers and products. Source: FDA Warning Letters; letters used the term “marketed unlawfully” to describe products that should not be marketed or sold.

From our initial sample of 100 retailers who had received FDA Warning Letters, 89 represented unique retailers and were included in the analysis (Figure 1). Of the total 89 FDA Warning Letters representing unique retailers (Supplement, Table S1), 29 (33%) named a single product as being “marketed unlawfully”, 25 (28%) listed 2 products, 18 (20%) listed 3 products, and the remaining 21 (24%) listed between 4 and 11 products. In total, the Warning Letters in our sample specifically named 227 products, and 165 (73%) of these products were sold online at the time each letter was issued.

### Retailer Locations

Of the 86 retailers located in the US, 41 (48%) were in the South, 17 (20%) in the West, 15 (17%) in the Northeast, and 13 (15%) in the Midwest. Of the three remaining retailers, one was in Canada and the other two did not provide contact information, meaning their location could not be identified.

### Types of Violations

The most common primary violation was the sale of new tobacco products without required marketing authorization, a statement made in 74 (83%) of FDA Warning Letters in our sample (Table 1). The second most common violation type was the failure to include the required nicotine warning statement: “This product contains nicotine. Nicotine is an addictive chemical.” The majority of Warning Letters 77 (87%) listed a single type of violation, the remainder listed between 2 and 5 primary violations.

**Table 1.** Violation Types Listed in Each FDA Warning Letter^a^ (#, %).

New Tobacco Products Without Required Marketing Authorization	74	83%
[Product] Fails to include the required nicotine warning statement	15	17%
Additional considerations	3	3%
Modified risk tobacco product violation	2	2%
A tobacco product without required ingredient listing submission	2	2%
Sales to minors violation	1	1%
A tobacco product not listed with FDA	1	1%
Tobacco products manufactured in an establishment not duly registered	1	1%
Failure to pay assessed user Fees	1	1%

^a^Violations were drawn from letters sent to 89 retailers; some retailers were notified of multiple primary violations.

### Remediation of Violations

Among the 89 retailers in our sample, the Warning Letter listed a website for 57 (64%) retailers (see Figure 1). At the time of our analysis, 10 (18%) of these 57 retailers had taken down their websites or made them inaccessible (password protected), leaving 47 (82%) of 57 retailers with websites that were still active.

All the FDA Warning Letters in our sample indicated that retailers must stop sales of the marketed unlawfully product or products within 15 days of receipt; at the time of our review, each retailer had had 3-18 months to remediate; as indicated above, we determined that the violation had been corrected if the retailers no longer listed the named product for sale. Among the 47 retailers included in the sample that had active websites at the time of analysis (Figure 1), 20 (43%) still listed a “marketed unlawfully” product identified in the FDA Warning Letter as available for purchase. (Retailers who receive Warning Letters may contest the notice of violations and if they do, the FDA website posts the response; there were no archived response letters in our sample.) For 14 (30%) of the 47 retailers, we were unable to determine whether the marketed unlawfully product was still available for sale. Examples included posting a photo of a display case with the product names too blurred to read, including photos with the product turned away from the camera to hide the label, or password-protecting the portion of the website where products could be purchased. Thirteen (28%) of the 47 retailers did not show marketed unlawfully products identified by the FDA on their websites. Among these 47 retailers, the FDA identified a total of 165 specific products that they had marketed unlawfully; of these 165 products, 57 (35%) were still available for sale at the time of this study (Figure 1).

### Processes in Place to Deter Access by Minors

Of the 47 retailers identified as selling products online, 32 (68%) had created a restriction on browsing related to age, while the remaining 15 (32%) did not attempt to restrict access to minors. Not all the limitations imposed on browsing were consistent with US laws limiting the sales of tobacco products to people who are at least 21 years old. Eighteen sites asked visitors to confirm they were at least 21 years old, the minimum age of legal access in the US at the time of analysis. Of the remaining 14 sites, 8 asked visitors to confirm that they were “legal age”, 2 asked visitors to confirm that they were at least 18 years old, 1 site asked visitors to confirm they were at least 19 years old, and 1 asked site visitors to enter their date of birth (which was not externally validated). The remaining 2 sites did not indicate a minimum age of legal access.

### Marketing Claims and Health Warnings

As described above, within the sample of 47 online retailers we identified, the FDA named 165 specific products as having been marketed unlawfully, and of those 165 products, 57 (35%) were still available for purchase at the time of the study (Figure 1). Of these 57 marketed unlawfully products, 12 (21%) included the required health warning, “This product contains nicotine. Nicotine is an addictive chemical.” A larger share of products made marketing claims intended to appeal to purchasers; 25 (43%) mentioned product flavors, 15 (26%) described the product’s taste, and 12 (21%) provided a strength descriptor. In contrast to earlier research findings and counter expectations, only one website made a claim about social acceptability, only one indicated that a product had been submitted for FDA approval, and only one mentioned the use of a product for cessation.

### Flavors

**Figure 2.**
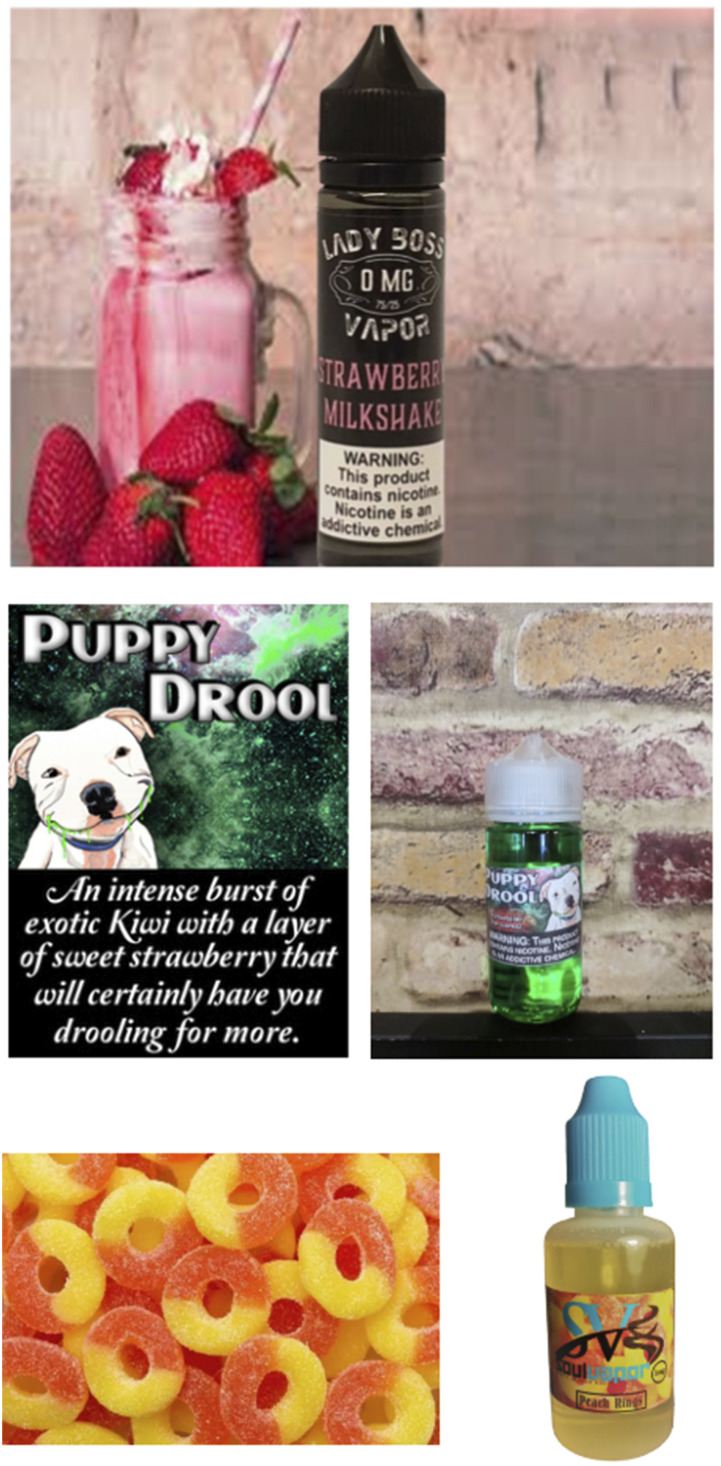
Examples of product marketing images. Top image: Lady Boss Strawberry Milkshake product photo showing nicotine solution bottle, strawberry milkshake, and strawberries taken from retailer website; Middle image: Puppy Drool product photos showing label information (L) and nicotine solution bottle (R) copied from retailer website; Bottom image: Soul Vapor Peach Rings product photos showing peach rings background from label and product page (L), and nicotine solution bottle (R), both copied from retailer website.

Our classification of flavors generated six major categories: alcohol, conceptual, food-related, locations, plants and animals, and tobacco; products that did not mention a flavor or that were labeled as unflavored were categorized as belonging to none of these categories (see Table 2). We classified all products listed in Warning Letters; and assessed whether each retailer sold one or more products identified by the FDA as problematic under each classification. Of the 89 retailers, 7 (8%) received warnings about products with names that involved alcohol (eg, Mojito, Scotch), 38 (43%) conceptual (eg, Beetlejuice, Extacy [sic], Kiss, Love, Smurf), 53 (60%) food-related (eg, Ambrosia, Apple, Chocolate, Cookie, Grahams, Gummy, Peach, Watermelon), 10 (11%) location (eg, Egypt, Texas), 16 (18%) plants and animals (eg, Bunny, Pitbull, Roses), and 2 (2%) tobacco. To better interpret the promotion of these products, particularly the products with conceptual names, we took screenshots of products still available for purchase; examples are provided in Figure 2.

**Table 2.** Flavor Terms and Categorization.

Categories	Terms
Alcohol	Brew, fireball, hop, mead, mojito, scotch
Food-related	Acai, ambrosia, apple, bakery, banana, berry, candy, cantaloupe, caramel, cereal, cherry, chocolate, cinnamon, coconut, con leche, cookie, cream pie, crunch, cucumber, custard, fruit, grahams, gummy, honey, horchata, jawbreaker, lemon, loops, mango, munch, orange, peach, rainwater, razz, succulent, vanilla, waffle, watermelon
Plants and animals (real)	Bunny, gorilla, menthol, mint, mocking bird, parrot, peppermint, pitbull, rose, wintergreen
Locations	Arctic, avalon, back country, bahama, Egypt, lost coast, salt lake, Texas, tropical, western
Tobacco	Tobacco
Concept	American, amulet, asgard, barn, bazoo, beetlejuice, black chapel, blast, blu-yo, boom, buckland, caliburn, citrine, classic, climax, coldwater, cowboy, crazy, doom, dragon, drool, duke, escapage, extacy, fireside, frosted, heisenberg, high noon, hobby, hypnozone, kiss, lonestar, love, lux, marilyn, mystic, nebulychee, ninja, pimp, skull, smurf, summit, sunrise, sunset, switch back, thor, trail head, unicorn, vineland, wayfarer, widow, yearn

Source: FDA Warning Letters coded by the authors.

Some FDA Warning Letters expressed concerns about marketing to minors due to flavor and packaging choices even if the named violation did not pertain directly to minors. In a Warning Letter written on July 20, 2020, to Vape Deal LLC, the listed violation was “New Tobacco Products Without Required Marketing Authorization.” The letter text stated “FDA finds the Strawberry Churrios by The Milkman ENDS product particularly concerning because this ENDS product (see Exhibit A) appears to imitate food products that are typically marketed toward and/or appealing to children. Specifically, the ENDS product packaging looks very similar to and features graphics/images of milk cartons (see Exhibit B), which are commonly marketed toward and/or appealing to children.”^43^ (Supplement, Figure S1)

## Discussion

Our findings suggest that after the onset of the COVID-19 pandemic, FDA Warning Letters focused primarily on violations related to the sale of products without premarket authorization. In 2018, the most common primary violations listed in Warning Letters had involved sales and/or marketing to minors, either individually or in combination with other violation types (98%).^36^ Past research on tobacco-related FDA Warning Letters issued in 2018 found that 17% of retailers failed to correct violations^36^; in contrast, 43% of retailers in our 2021-2022 sample had failed to correct violations. Overall, in the first two years of the COVID-19 pandemic, we found that the FDA was more likely to focus on PMTA violations in communicating with online tobacco retailer and that these retailers were less likely to correct violations identified by the FDA, suggesting that the effectiveness of enforcement declined.

Consistent with research suggesting that a large share of tobacco products used by minors were purchased online,^34,35^ our findings also demonstrated that few retailer websites had meaningful processes in place that would deter youth browsing. Almost one-third of online tobacco retailers that the FDA identified as selling products in violation of existing regulations did not screen for age on their websites. Fewer than four in ten retailers who had received Warning Letters correctly identified the minimum age of legal access in the US as being 21 years. Furthermore, all but one retailer website with an age screener required only that visitors click a button stating they were old enough to purchase tobacco products. Notably, the retailers in this sample had already been notified by the FDA that they had marketed tobacco products in violation of existing law. Ideally, the awareness that the FDA was monitoring retailers would lead them to develop additional processes that would deter browsing by minors. These findings suggest a need for additional research on a broader sample of online retailers, and the value of developing standards for age-gating that could be required of online tobacco retailers.

Our study found that the majority of marketed unlawfully flavored products listed in FDA Warning Letters for 2020-2021 referenced food, and most of these food-related products referenced flavors that appeal to children; examples included gummies, “booberry”, grahams, and jawbreakers.^19,22^ The results of this analysis suggest that marketing by online retailers has moved away from earlier claims that alternative tobacco products could be used for smoking cessation, were less expensive than combustible cigarettes, and could be used in places where smoking was banned.^1-10^

### Strengths and Limitations

Our analysis was conducted over the course of four months in 2022, and retailers may have changed their marketing over time. Similarly, due to issuance of FDA Warning Letters at different times, some retailers had more time to remediate. Our reliance on FDA Warning Letters meant that identified violations reflected FDA resources and regulatory priorities; as a result, we were unlikely to identify all retailers that violated regulations. In addition, the available of some products could not be determined through online searches when Warning Letters were issued to physical stores that did not list an online presence or list products. However, our methods have been used and validated in prior research.^36^ We did not undertake actual purchases of products, making it impossible to assess age verification upon point of sale or delivery; this limitation is consistent with previous research. Future research could expand on this work by conducting research on a broader sample of online retailers, to determine whether these marketing practices are consistent even among those not identified by the FDA. Future research could also consider whether the identification of more retailers with violations in the US South exacerbates existing health inequities; smoking rates in the US South and Midwest are higher than in other regions, leading to lower life expectancies, and these regions have fewer policy protections related to tobacco use such as prevention and cessation programs, smokefree policies, and flavor bans.^44^

With respect to shifts in the types of violations, marketing authorization was a new requirement that did not exist at the time of prior research; the shift to this violation does not indicate that marketing to minors had been resolved, given that Warning Letters specifically indicated that products that appealed to minors were problematic. Instead, the FDA may have redirected limited existing resources to pursue binary violations (e.g., authorized or not authorized) that were easier to prove than violations that require interpretation such as marketing to minors, or both.

### Conclusions

Our findings provide new information regarding shifts in retailer marketing of alternative tobacco products in ways that appealed to youth at the onset of the COVID-19 pandemic, a period when all purchasing shifted online, and that likely increased youth access to these products given the limited efforts to establish processes that would deter youth browsing. Given the shift to online purchasing of tobacco products after the COVID-19 pandemic, the extensive promotion of flavored tobacco products that appeal to minors, and the fact that retailer failures to remediate violations have increased, the FDA should consider expanding enforcement of existing regulations on tobacco products marketed unlawfully. The discovery that retailers made little effort to prevent underage access to websites also suggests the value of creating standards for age-gating for online retailers who sell products intended for adult use such as tobacco. These interventions may help reduce the initiation of tobacco product use, given that online tobacco marketing involves multiple flavors known to appeal to young people.

## Supplemental Material

Supplemental Material - An Observational Study of Regulatory Violations Related to Online Tobacco Product Marketing and Retailer Responses to US FDA Warning Letters Supplemental Material for An Observational Study of Regulatory Violations Related to Online Tobacco Product Marketing and Retailer Responses to US FDA Warning Letters by Dorie E. Apollonio, Cathi E. Dennehy, Candy Tsourounis, and Tanner Wakefield in Tobacco Use Insights.
