# Supplementary material for: An Observational Study of Regulatory Violations Related to Online Tobacco Product Marketing and Retailer Responses to US FDA Warning Letters
Source: Tob Use Insights. 2024 Nov 13;17:1179173X241300825. doi: 10.1177/1179173X241300825 (PMC11561981; doi:10.1177/1179173X241300825)
Supplement: Supplemental Material - An Observational Study of Regulatory Violations Related to Online Tobacco Product Marketing and Retailer Responses to US FDA Warning Letters [file sj-pdf-1-tui-10.1177_1179173X241300825.pdf]

An observational study of regulatory violations related to online tobacco product marketing and retailer responses to US FDA Warning Letters

Appendix

Figure S1: Images from FDA Warning Letter to Vape Deal LLC

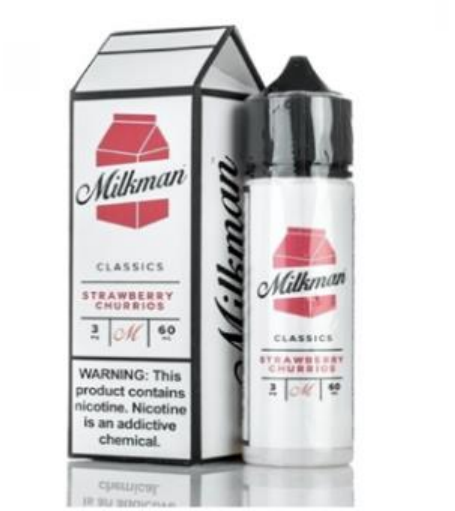

Exhibit A

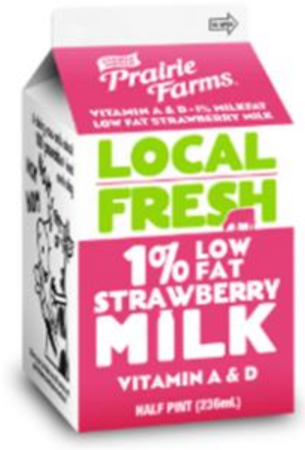

Exhibit B

Table S1: List of FDA Warning Letter IDs for sample

MARCS-CMS 597094
MARCS-CMS 604548
MARCS-CMS 604870
MARCS-CMS 605075
MARCS-CMS 605635
MARCS-CMS 605716
MARCS-CMS 605736
MARCS-CMS 605739
MARCS-CMS 606730
MARCS-CMS 606980
MARCS-CMS 607362
MARCS-CMS 607458
MARCS-CMS 607609
MARCS-CMS 607938
MARCS-CMS 608062
MARCS-CMS 608199
MARCS-CMS 608385
MARCS-CMS 608399
MARCS-CMS 608452
MARCS-CMS 608457
MARCS-CMS 608526
MARCS-CMS 608554
MARCS-CMS 608556
MARCS-CMS 608863
MARCS-CMS 609065
MARCS-CMS 609142
MARCS-CMS 609573
MARCS-CMS 610020
MARCS-CMS 610634
MARCS-CMS 610648
MARCS-CMS 612327
MARCS-CMS 612794
MARCS-CMS 612929
MARCS-CMS 613129
MARCS-CMS 613225
MARCS-CMS 613229
MARCS-CMS 613229
MARCS-CMS 613471
MARCS-CMS 613503
MARCS-CMS 613506
MARCS-CMS 613545

MARCS-CMS 613545
MARCS-CMS 613735
MARCS-CMS 613738
MARCS-CMS 613969
MARCS-CMS 613982
MARCS-CMS 614115
MARCS-CMS 614158
MARCS-CMS 614196
MARCS-CMS 614210
MARCS-CMS 614222
MARCS-CMS 614299
MARCS-CMS 614673
MARCS-CMS 614881
MARCS-CMS 614935
MARCS-CMS 615109
MARCS-CMS 615115
MARCS-CMS 615203
MARCS-CMS 615519
MARCS-CMS 615960
MARCS-CMS 616094
MARCS-CMS 616217
MARCS-CMS 616348
MARCS-CMS 616374
MARCS-CMS 616381
MARCS-CMS 616903
MARCS-CMS 617222
MARCS-CMS 617265
MARCS-CMS 617443
MARCS-CMS 617946
MARCS-CMS 618195
MARCS-CMS 618272
MARCS-CMS 618425
MARCS-CMS 618809
MARCS-CMS 618821
MARCS-CMS 618907
MARCS-CMS 619171
MARCS-CMS 619305
MARCS-CMS 619418
MARCS-CMS 619841
MARCS-CMS 620717
MARCS-CMS 620749
MARCS-CMS 621220
MARCS-CMS 621721
MARCS-CMS 622333

MARCS-CMS 622895
MARCS-CMS 623313
MARCS-CMS 623627
None provided (BloVape; January 29, 2021)
